# Understanding Molecular Actors of SARS-CoV-2 Virulence to Tackle COVID-19 Outbreak

**DOI:** 10.3390/cells11223597

**Published:** 2022-11-14

**Authors:** Rita Berisio

**Affiliations:** Institute of Biostructures and Bioimaging, IBB, CNR, Via Pietro Castellino 111, I-80131 Naples, Italy; rita.berisio@cnr.it

COVID-19 (coronavirus disease 2019) is a threatening disease caused by the novel enveloped, positive-sense, single-stranded RNA beta-coronavirus, denoted as SARS-CoV-2. In March 2020, COVID-19 was declared as a pandemic by the World Health Organization (WHO), given the high number of cases and the spread of the disease worldwide. SARS-CoV-2 shares 79.5% of its genetic code with the virus SARS-CoV, which was identified in 2002 as the causative agent of the SARS pandemic. In a similar manner to the causative agent of SARS, SARS-CoV-2 causes severe respiratory problems, including cough, fever, and respiratory difficulties. The scientific community has heavily focused on accelerating research by identifying molecular actors of SARS-CoV-2 virulence and infection, which are key in the delivery of therapeutics to hamper vital functions of viral replication.

A key step of the SARS-CoV-2 life cycle is the replication of its viral genome within the infected cells [1]. This is a complex process that involves the action of several viral and host proteins that perform RNA polymerization, proofreading and final capping. Romano et al. provide an update of the structural and functional data on the key actors of the replicatory machinery of SARS-CoV-2 to fill the gaps in the currently available structural data. Moreover, learning from similar viruses, the authors reconstruct the pattern of interactions among the protein actors of the SARS-CoV-2 RNA polymerase machinery [1].

Another factor that is mandatory for SARS-CoV-2 infection is the viral access to the host cells through the Spike protein. As it is essential for infection, the spike protein has been exploited as a target for vaccine development [2], functioning as a target of the neutralizing antibodies and interactors [3,4,5]. Hatmal et al. performed an in-depth review of the literature, covering the structural and mutational aspects of the spike protein in the context of SARS-CoV-2 and compared these with those of SARS-CoV and Middle East respiratory syndrome CoV (MERS-CoV) [6]. Their analytical approach consisted of an initial genome and transcriptome analysis, followed by primary, secondary and tertiary protein structure analysis. Additionally, they investigated the potential effects of these differences on spike protein binding and interactions to angiotensin-converting enzyme 2 (ACE2), and deduced that SARS-CoV-2 and SARS-CoV use different regions of the spike protein receptor-binding motif and different binding modes to ACE2 [6]. Similarly, Papageorgiou et al. reviewed the current structural knowledge of the SARS-CoV-2 spike glycoprotein and its complexes with ACE2 and antibodies, which is key in the development of therapies and vaccines against the new coronavirus [7]. Consistently, the spike protein is used as the antigen of both mRNA and adenovirus-based vaccines licensed for use [8]. Emerging variants are being identified around the globe, and there is, therefore, emerging evidence of reduced neutralization of some SARS-CoV-2 variants [8].

A possible role of furin and osteopontin in SARS-CoV-2 disease, especially in patients with diabetes, is proposed by Adu-Agyeiwaah et al. [9]. Furin is a proprotein convertase implicated in the proteolytic processing and activation of several precursors, including the SARS-CoV-2 spike protein. Its overexpression is associated with an increased risk of diabetes-linked premature mortality and is regulated by osteopontin, a cytokine-like phosphoglycoprotein, whose levels are elevated in diabetic patients. Since furin plays an important role in promoting SARS-CoV-2 entry and replication in the host cell, the authors propose that diabetes-induced osteopontin and furin upregulation results in worse outcomes in diabetic patients with SARS-CoV-2 infection. Thus, targeting the osteopontin–furin axis may be a plausible strategy for reducing mortality in SARS-CoV-2 patients with diabetes [9]. 

Immunomodulatory mechanisms play a significant role in SARS-CoV-2 infection, as they may cause a severe syndrome in COVID-19 patients called “cytokine storm”. Kreis et al. summarize studies regarding the potential evidence for trans-placental transmission of SARS-CoV-2, as viral infections may predispose pregnant women to a higher rate of pregnancy complications [10]. Potential molecular mechanisms are proposed, whereby the placenta serves as a defense front against pathogens by regulating the interferon type III signalling, microRNA-triggered autophagy and the nuclear factor-B pathway [10]. Therefore, the placenta may mitigate violent immune responses, possibly minimizing cell and tissue damage [10]. Immunomodulation in COVID-19 patients is also discussed by Parackova et al., who present a detailed dissection of functional profiles of neutrophils, dendritic cells, and monocytes in COVID-19 patients [11]. They highlight the crucial role of neutrophils as drivers of hyperinflammation associated with COVID-19 disease via the shift towards their immature forms, enhanced degranulation, cytokine production, and augmented interferon responses [11]. The role of reaction cycles of halogen species in immune defense against SARS-CoV-2 is discussed by Lu, who proposes a class of halogenated aromatic drugs as possible therapeutics [12]. In addition, Cappellano et al. propose circulating platelet-derived extracellular vesicles (EVs), a hallmark of SARS-CoV-2 infection, as biomarkers for rapid diagnostic tests on unmanipulated blood samples [13]. 

Taken together, the data reported in this Special Issue offer an open-access forum that aims to address multi-disciplinary approaches to understand and challenge the COVID-19 outbreak and possible long-term effects.
